# Linking air pollution appraisal to EFL teachers' negative emotion via mental effort: the moderating role of working memory capacity

**DOI:** 10.3389/fpsyg.2025.1521597

**Published:** 2025-02-26

**Authors:** Yijun Shi, Shuhua Wang, Qi Hao

**Affiliations:** ^1^College of Foreign Languages and Cultures, Beijing Wuzi University, Beijing, China; ^2^The School of Information Resource Management, Renmin University of China, Beijing, China

**Keywords:** EFL teacher emotions, air pollution appraisal, mental effort, cognitive load theory, working memory capacity

## Abstract

Based on Cognitive Load Theory, this study developed a moderated mediation model to examine the relationship between English as foreign language (EFL) teachers' air pollution appraisal and negative emotions. Specifically, it hypothesizes that air pollution appraisal significantly increases the mental effort of EFL teachers, which in turn leads to the manifestation of negative emotions. Additionally, the study introduces the concept that the working memory capacity of EFL teachers can negatively moderate the impact of increased mental effort on their emotions, effectively attenuating the overall mediating effect. Data for this research was gathered from daily diary surveys of 182 EFL teachers across 23 high schools in Shanxi Province, China. The hypotheses were tested using two-level Hierarchical Linear Modeling and Monte Carlo analysis, with all proposed hypotheses receiving empirical support. This study significantly enriches the existing literature on air pollution appraisal, EFL teacher emotion, and Cognitive Load Theory, offering crucial practical insights for educational institutions on how to mitigate the adverse effects of air pollution on teachers.

## 1 Introduction

Over the past several decades, alongside the rapid advancement of industrialization, air pollution has emerged as a significant global challenge. According to data from the World Health Organization (WHO), an alarming 92% of the world's population lives in cities where air quality fails to meet established standards (Fehr et al., 2017). Research indicates that air pollution can have a severe impact on individuals' physical health, contributing to respiratory issues, lung diseases, and cardiovascular disorders (e.g., Schlenker and Walker, 2016; Irfan, 2024; Wu et al., 2024). In addition to its detrimental effects on physical health, studies have increasingly shown that air pollution can also influence people's negative behaviors and emotional wellbeing. For example, it may undermine pro-environmental behaviors (Ming et al., 2022), disrupt consumer consumption patterns (Liu et al., 2022), and even have a detrimental impact on mental health (Wang et al., 2024). As a result, understanding the effects of air pollution on both physical and mental health, particularly its influence on emotions, has become a critical area of focus within environmental psychology research.

Although the academic community has made notable progress in exploring the relationship between air pollution and individual emotions, a careful review of the literature reveals several significant gaps. First, most existing research primarily focuses on objective measures of air pollution, often overlooking the impact of individuals' perceptions of pollution. Previous studies have demonstrated that the effect of perceived “stimuli” can differ significantly from that of the stimuli themselves (Fehr et al., 2017). Second, while research on air pollution's effects on behavior and emotions has largely concentrated on fields such as business administration and marketing (e.g., Khan et al., 2024; Li et al., 2024; Liu et al., 2022), there is comparatively little research within the domains of education and teaching, particularly in the area of language learning. Although scholars in education have acknowledged the importance of teachers' emotions (e.g., Chen, 2021; Han et al., 2023), few have examined the role of the natural environment in shaping these emotions. For instance, many studies still focus on the physical health effects of air pollution on teachers (Lipsett et al., 2011), rather than considering its emotional impact. Finally, much of the existing literature on the relationship between environmental pollution and emotions tends to focus on the direct effects of pollution (e.g., Palma et al., 2022; Shin et al., 2018), often neglecting potential underlying mediating mechanisms and moderating factors that may shape this relationship. This narrow focus limits the understanding of the complex ways in which air pollution influences emotional responses.

Here, to bridge the aforementioned research gaps, we have crafted a cohesive model which synthesizes Cognitive Load Theory (Sweller et al., 1998), insights from the psychology of natural environments (Fehr et al., 2017), and literature on teacher emotions (Han et al., 2023). We propose that EFL teachers' perception of air pollution can lead to the emergence of negative emotions. The underlying mechanism of this effect is rooted in the continuous increase in cognitive load. According to Cognitive Load Theory, perceiving air pollution introduces an extraneous cognitive load, requiring teachers to allocate additional mental resources and exert greater mental effort. Given the relatively fixed capacity of individuals' mental resources, this situation can trigger cognitive overload, resulting in negative emotions such as anxiety and depression. To better understand the dynamic characteristics of how air pollution assessment impacts EFL teachers' emotions, we have also considered the role of individual cognitive abilities. Prior studies suggest that variations in cognitive abilities can influence responses to cognitive load (De Melo et al., 2020). Accordingly, we have introduced Working Memory Capacity (WMC) as a moderating factor to explore how the aforementioned mechanism differs among individuals with varying levels of WMC. Furthermore, to effectively mitigate the potential common method bias inherent in traditional cross-sectional studies and to gain a deeper understanding of the relationship between air pollution assessment and the cognitive and emotional changes of EFL teachers (Barnes et al., 2015), we conducted a 10-day daily diary study with 182 EFL teachers from urban areas in China to test the proposed model.

In total, our study makes several contributions. Firstly, our study highlights the impact of English teachers' subjective assessment of air pollution on their emotions within the field of language teaching, rather than solely focusing on the objective reality of air pollution. This perspective significantly broadens the scope of existing literature on the relationship between air pollution and emotional wellbeing. Secondly, we extend the application of Cognitive Load Theory by proposing that EFL teachers' daily assessments of air pollution contribute to an increased cognitive burden. This heightened cognitive load, in turn, escalates mental effort and triggers negative emotions. By employing Cognitive Load Theory, we delve into the fundamental dynamics underpinning this relationship. Thirdly, our research identifies a critical boundary mechanism that moderates the relationship between air pollution appraisal and emotions at the individual level. We demonstrate that higher individual WMC can effectively mitigate the negative emotional impact of air pollution assessment on teachers, providing valuable insights into individual differences in emotional resilience.

## 2 Literature review and hypotheses development

### 2.1 Air pollution appraisal

Air pollution has emerged as a global challenge, largely due to the swift pace of industrialization and urbanization (Fehr et al., 2017). Over the past three decades, China has experienced remarkable economic growth. However, this prosperity, heavily dependent on fossil fuels, has come at the cost of significant environmental degradation (Lu et al., 2020). The 2023 Bulletin of the State of China's Ecological Environment (Ministry of Ecology Environment of the People's Republic of China, 2024) reveals a concerning statistic: ~40% of the 339 prefecture-level cities in China are still grappling with air quality that does not meet the National Ambient Air Quality Standards (NAAQS). Among the various pollutants, non-compliance with fine particulate matter (PM_2.5_) standards stands out as the most pressing air pollution concern.

The preponderance of air pollution research is dedicated to understanding its ramifications on individual health. Researchers underscore that exposure to polluted air, whether chronic or acute, can trigger a wide array of health problems (Braithwaite et al., 2019). These include respiratory ailments, lung tissue damage, cardiovascular stress, congenital disorders, developmental impediments, and weakened immune function (Kampa and Castanas, 2008). Recently, the field of organizational psychology has increasingly acknowledged the critical impact of air pollution on employee work behavior, attitudes, and mental wellbeing. Studies have suggested that exposure to polluted air may correlate with an increase in counterproductive work behavior and a decrease in organizational citizenship behavior (Fehr et al., 2017). Additionally, there is evidence suggesting that air pollution can induce anxiety among employees, potentially leading to an increase in unethical conduct (Gong et al., 2020). Moreover, research has indicated a positive link between air pollution and certain leadership approaches, such as abusive supervision and laissez-faire management styles (Khan et al., 2024). Importantly, these studies mainly focus on individuals' subjective perceptions of air quality, that is, air pollution appraisal, rather than objective measurement data. Experts believe that the impacts of air pollution on behavior and psychology are closely related to individuals' subjective assessments of air pollution within specific temporal and spatial contexts (Fehr et al., 2017). Consequently, there is a growing preference among researchers to investigate the impacts of air pollution appraisal, moving beyond a reliance on purely quantitative air pollution metrics.

While there has been some progress in researching the relationship between air pollution and individuals, related studies in the field of education remain limited. Experts in education and linguistics have noted the harmful effects of air pollution on both teachers' and students' health, as well as on teaching effectiveness. For instance, Ostro et al. (2010) reported that prolonged exposure to fine particulate matter could elevate mortality rates among teachers. Similarly, Lipsett et al. (2011) posited that such exposure could detrimentally impact teachers' cardiovascular and respiratory health. Furthermore, studies have revealed that air pollution can erode students' academic performance (Gartland et al., 2022). For example, reduced teacher attendance due to air pollution has been linked to significant declines in children's reading and math scores (Balakrishnan and Tsaneva, 2021). In the realm of language education, recent research has utilized English proficiency exams of Chinese university students as a backdrop, suggesting that short-term exposure to PM_2.5_ can adversely affect the cognitive capacities of language learners (Yao et al., 2023). These research findings have been instrumental in stimulating and directing scholarly inquiry into the effects of air pollution on educational performance in educational and linguistic disciplines. Nevertheless, to our awareness, the specific influence of teachers' perceptions of air pollution on their emotional states within language education has not been thoroughly explored.

### 2.2 EFL teacher emotion

Emotion is a multifaceted concept without a universally accepted definition, with three dominant perspectives shaping its study: the psychological view, which considers emotions as internal states; the social constructionist perspective, which emphasizes interpersonal interactions; and the interactionist approach, which integrates individual traits and social relationships (Chen, 2021; Han et al., 2023). The interactionist perspective, increasingly influential in teacher emotion research, defines teacher emotions as internal sensations shaped by interactions with students, colleagues, parents, and environments (Farouk, 2012). In terms of classification, teacher emotions are typically categorized as positive (e.g., enjoyment, pride) or negative (e.g., anxiety, fear). While this binary system has faced criticism for oversimplification, Parrott's multi-tiered model offers a more detailed approach, distinguishing between basic and complex emotions but still adhering to the positive-negative dichotomy (Chen, 2019). This study adopts the binary classification for its clarity and widespread acceptance.

As inquiry into teacher emotions progresses, there is a growing scholarly interest in the emotional landscape within specialized academic domains, such as English language instruction (Han et al., 2023). EFL teachers, in comparison to their counterparts in fields like science, mathematics, and history, face unique emotional challenges due to the heightened anxiety and discomfort that can accompany teaching a non-native language (Lee and Lew, 2001). Initial studies on the emotions of EFL teachers concentrated on anxiety, particularly the phenomenon known as language teacher anxiety (Horwitz, 1996). These investigations often employed qualitative techniques to delve into the feelings of unease, apprehension, and discomfort that non-native English teachers might encounter (Xu, 2018). They also examined how these emotional states impacted teaching effectiveness, pedagogical strategies, student emotions, and the teachers' own wellbeing (Horwitz, 1996). In recent years, research on the emotions of EFL teachers has evolved to become more expansive and sophisticated. It has expanded beyond merely language anxiety to encompass a broader range of emotions and has employed more rigorous and scientific research methodologies, including quantitative methods or a combination of quantitative and qualitative approaches (Xu, 2018).

So far, research on EFL teacher emotions has predominantly been approached from cognitive, sociocultural, and linguistic viewpoints (Han et al., 2023). The current literature on EFL teacher emotions primarily focuses on the content of these emotions, their functional effects, and the underlying causes. In terms of content, studies have concentrated on categorizing EFL teacher emotions into positive and negative groups (Gallo and Tassinari, 2017), and exploring emotional competencies such as emotional regulation (Talbot and Mercer, 2018), emotional intelligence and emotional labor (Kang, 2022). Research on the impacts of EFL teacher emotions has explored their multifaceted effects, specifically: (a) on the teachers themselves, influencing their educational beliefs (Barcelos, 2015), professional identity (Yang et al., 2021), and sense of wellbeing (Derakhshan et al., 2022); (b) on their students, affecting levels of engagement (Alvandi et al., 2015) and academic performance (Pourbahram and Hajizadeh, 2018); and (c) on teaching activities, impacting teaching effectiveness (Nikoopour and Esfandiari, 2017) and teaching methods (Kirmizi and Sariçoban, 2020). The research on the antecedents of EFL teacher emotions continues to be a subject of active scholarly debate, presenting a diverse array of viewpoints. Some researchers emphasize the importance of contextual elements, such as cultural nuances (Chahkandi et al., 2016), classroom dynamics (Li et al., 2021), and institutional policies (Nazari and Molana, 2023), in shaping the emotional landscape of EFL teachers. Conversely, others argue that individual traits of the teachers themselves, such as personality (MacIntyre et al., 2019), are fundamental in giving rise to these emotions. Additionally, a subset of scholars highlights the significant role of interpersonal relationships with colleagues, leaders, and students in influencing EFL teacher emotions (Taxer et al., 2019). Recent research has called for an in-depth exploration of the complex interplay between cognition and emotions among EFL teachers, suggesting that recognizing and addressing this link is crucial for leveraging the power of teacher emotions to enhance pedagogical skills and advance professional development (Shi, 2021).

### 2.3 Cognitive Load Theory

The central tenet of Cognitive Load Theory is that the cognitive resources available in working memory are pivotal for individuals to effectively perform specific tasks (Sweller et al., 1998). Given the finite capacity of working memory, the amount of cognitive resources one can muster is inherently constrained. Consequently, attention diverted to irrelevant elements may detract from the mental resources that should be dedicated to the core task, thus escalating an individual's cognitive load (Feldon, 2007). Cognitive Load Theory delineates three principal sources of cognitive load: intrinsic, extraneous, and germane (Paas et al., 2016). Intrinsic load stems from the complexity of the task and an individual's capacity to process the information presented. Extraneous load originates from the instructional design or presentation that imposes additional, non-essential cognitive demands on working memory, often manifesting as internal or external distractions. Conversely, germane load is beneficial, representing the cognitive resources devoted to constructing meaningful schemas during task completion (Sweller, 2010). According to Cognitive Load Theory, the aggregate of these three loads should be balanced to not surpass the available mental resources for task execution (Sweller, 2010). Task failure frequently occurs when excessive extraneous load or overwhelming intrinsic load surpass an individual's capacity to effectively manage cognitive demands.

Cognitive Load Theory has been widely applied across various fields. In education, it informs curriculum design (Sweller, 2023), while in product development, it guides the creation of user-friendly software (Sevcenko et al., 2023). In this article, we apply this theory to explore how the assessment of air pollution impacts the emotions of EFL teachers. Air pollution, as a complex environmental stimulus, demands significant cognitive resources for perception and evaluation. When English teachers observe poor air quality during their daily interactions with the outdoor environment, their brains engage in assessing factors such as pollution levels and potential hazards, thereby increasing intrinsic cognitive load. Simultaneously, concerns about the potential health risks to themselves and other people they care about compel them to seek and process additional information, such as protective measures, further escalating extraneous cognitive load. Prolonged exposure to such high cognitive demands depletes the mental resources needed for emotional regulation. Similar to a computer overwhelmed by running too many programs, teachers struggle to manage their emotions effectively, leading to the emergence of negative emotions. In the next section, we will delve deeper into the aforementioned impact mechanism.

### 2.4 Linking air pollution appraisal to EFL teacher emotion through mental effort

Drawing on Cognitive Load Theory (Sweller et al., 1998), this study suggests that EFL teacher's air pollution appraisal will affect their negative emotion through mental effort. Mental effort, a construct rooted in Cognitive Load Theory (Sweller et al., 1998), pertains to the portion of cognitive capacity actively engaged to satisfy the demands of a given task (Paas and Van Merriënboer, 1994). This concept is a critical component of cognitive load, which also includes mental load—indicating an individual's perception of task difficulty—and performance, signifying the outcome of task execution (Paas and Van Merriënboer, 1994). According to Paas (1992), mental effort is considered a key variable for quantifying the actual cognitive load experienced. In cognitive load studies, mental effort is considered a more revealing indicator than other dimensions, offering deeper insights into the cognitive engagement required by tasks. Thus, some scholars equate the measurement of cognitive load with that of mental effort, treating cognitive load as an index of the mental effort expended (Feldon, 2007). This perspective sees cognitive load as a reflection of “the number of non-automatic elaborations in working memory necessary to solve a problem” (Feldon, 2007, p. 125).

According to Cognitive Load Theory (Sweller et al., 1998), we suggest that air pollution may exacerbate both intrinsic and extraneous cognitive loads for EFL teachers, thereby consuming the cognitive resources necessary for English language instruction and escalating their mental effort. The rationale is twofold: firstly, air pollution can adversely affect the cognitive abilities and respiratory health of EFL teachers. Research indicates that exposure to air pollutants, such PM_2.5_, may enlarge brain regions involved in cognitive processing, leading to reduced cognitive function (Li et al., 2024). Additionally, air pollution can cause respiratory distress (Lipsett et al., 2011). Given the cognitive demands of language comprehension and expression in English instruction (Kasap, 2021), teachers in polluted settings may struggle to effectively conduct language teaching tasks due to diminished cognitive capacities or physical discomfort, thus requiring increased mental effort to achieve educational goals. Secondly, assessing air quality can serve as a cognitive distraction for teachers. This process involves a complex cognitive effort to understand and interpret the sources, characteristics, and consequences of pollution, as well as its implications for students and the educational setting. This additional task can increase the extraneous cognitive load during English teaching, thereby heightening the mental effort required.

When EFL teachers need to increase their mental effort due to the challenges posed by air pollution, it can lead to intense negative emotions. On one hand, this heightened mental effort may directly trigger negative emotional responses. Air pollution requires EFL teachers to invest more cognitively to meet their instructional duties, intensifying the mental effort needed. This heightened effort can produce substantial cognitive dissonance, thereby evoking adverse emotional states such as anxiety, discomfort, and stress (Joormann and Quinn, 2014). Empirical research corroborates this perspective, demonstrating that an escalated cognitive load can markedly diminish an individual's positive emotional experiences (Guo et al., 2022). On the other hand, an upsurge in mental effort for teaching can undermine a teacher's capacity for emotional regulation, hindering the control and neutralization of negative emotions. Cognitive Load Theory stipulates that an individual's cognitive resource is finite (Sweller et al., 1998). As the mental effort of an EFL teacher escalates, so does the strain on their working memory, resulting in a diminished reserve of cognitive resources available for emotional regulation. Insufficient cognitive resources, as suggested by prior studies, can impede teachers' emotional regulation, leading to an increased prevalence of negative emotions due to difficulties in managing their own feelings (Talbot and Mercer, 2018).

Taken together, this study posits that air pollution appraisal augments the mental effort of EFL teachers, attributable to a decline in cognitive faculties and a degradation in physical wellbeing. This intensified mental effort subsequently impairs their capacity for emotional regulation, leading to an amplification of negative emotional reactions. Accordingly, we propose:

*H1*: EFL teacher's air pollution appraisal positively affects their negative emotion through mental effort.

### 2.5 The moderating role of working memory capacity (WMC)

Beyond examining the underlying mechanisms linking air pollution appraisal to negative emotions among EFL teachers, this study aims to clarify the boundary conditions that define the scope and strength of this relationship. With a focus on individual differences and cognitive load perspective, the research will investigate WMC as a potential moderator. Working memory is intricately linked to an individual's short-term memory system, serving as a cognitive framework that supplies essential information for ongoing cognitive tasks (Wilhelm et al., 2013). Therefore, WMC can be conceptualized as a construct that captures individual differences, specifically referring to “the ability to sustain goal-relevant information processing in the presence of alternative goals or other distractions” (Schmeichel et al., 2008, p.1527). This capacity is a pivotal concept within cognitive science, and scholars have explored its intricacies from various perspectives over the past three decades. For example, from the executive-attention perspective (Engle, 2002), WMC involves managing and sustaining focus amidst interference. The two-component theory views it as a combination of primary and secondary memory (Unsworth et al., 2009). Furthermore, WMC is also seen as a system for creating and maintaining arbitrary associations rapidly (Wilhelm et al., 2013). The measurement of WMC involves various techniques, but the operation span (OSPAN) task is the most commonly used method (Schmeichel et al., 2008). This approach combines mathematical computations with short-term memory tests, assessing an individual's capacity to recall target words while conducting mathematical operations, thereby gauging working memory proficiency.

WMC is closely linked to the Cognitive Load Theory, exerting a direct impact on the cognitive resources allocated to various cognitive tasks (Chen et al., 2018). As such, contemporary research on WMC focuses on its relationship with a range of cognitive functions, including resistance to interference (Conway et al., 2001), cognitive control (Kane and Engle, 2003), effects of cognitive load (Chen et al., 2018), collaborative learning (Du et al., 2022), driving performance (Broadbent et al., 2023), and creativity enhancement (Gong et al., 2023). In the field of emotion, research exploring the interplay between emotions and WMC has traditionally focused on how negative emotions like stress and anxiety impact WMC (e.g., Beilock and Carr, 2005). However, recent studies are shifting focus to the reciprocal influence of WMC on emotions. Notably, evidence suggests a negative correlation between WMC and an individual's ability to regulate emotions (Schmeichel et al., 2008). Those with higher WMC often show better emotional regulation skills, allowing them to more effectively manage their negative emotional responses (Schmeichel and Demaree, 2010).

Based on the above literature, we posit that WMC of EFL teachers can mitigate the impact of mental effort on their negative emotions. According to Cognitive Load Theory (Sweller et al., 1998), an increase in the cognitive resources needed to complete a task elevates mental effort. Given the finite nature of working memory, higher mental effort often results in increased stress, anxiety, and discomfort. Consequently, the extent of an individual's WMC might affect how mental effort influences negative emotions. For EFL teachers with higher working memory capacities, even though a rise in mental effort consumes some cognitive resources, their larger memory capacity ensures a substantial reserve of cognitive resources. This surplus can help manage other tasks, potentially reducing the occurrence of negative emotions. In other words, greater WMC can offset the adverse effects of increased mental effort, leading to fewer negative emotions. Thus, we propose:

*H2:* EFL teacher's working memory capacity moderates the positive relationship between mental effort and negative emotion, such that when EFL teachers have higher working memory capacities, this relationship is relatively weaker.

**Figure 1:**
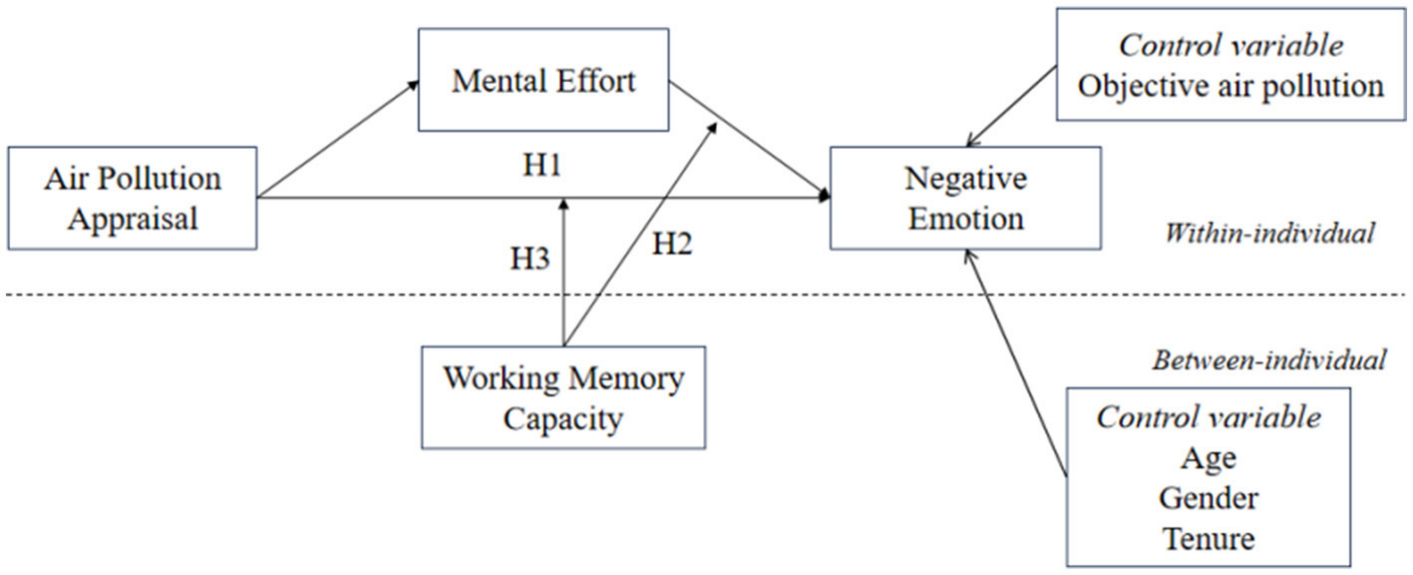
Theoretical model.

Drawing on methodologies from previous research (Edwards and Lambert, 2007), our study melds the mediating and moderating effects to develop a sophisticated moderated mediation model (see Figure 1). In this model, when EFL teachers perceive severe air pollution, their cognitive load increases, necessitating higher mental effort to complete teaching tasks. This increased mental effort elevates cognitive stress and diminishes emotional regulation abilities, leading to heightened anxiety and other negative emotions. However, if an EFL teacher has higher WMC, they possess ample cognitive resources to mitigate the adverse effects of increased mental effort resulting from their assessment of air pollution. Thus, teachers with greater WMC experience fewer negative emotions compared to their counterparts with lesser capacity. This suggests that the WMC of EFL teachers conditionally moderates the “air pollution appraisal–mental effort–negative emotion” relationship. Therefore, we propose:

*H3:* EFL teacher's working memory capacity moderates the mediating relationship between air pollution appraisal and negative emotion via mental effort, such that when EFL teachers have higher working memory capacities, this relationship is relatively weaker.

## 3 Method

### 3.1 Research context

Given our research focus on the influence of air pollution on teachers' emotional wellbeing, it is crucial to select regions with notably high air pollution levels. Air quality in Northern China is significantly worse compared to the Southern regions. Previous research indicates that the life expectancy of residents in Northern Chinese cities is, on average, 5.5 years shorter than that of their Southern counterparts, primarily due to the detrimental effects of air pollution (Qian et al., 2016). Consequently, we choose Shanxi Province in Northern China as the focal point of our research for several compelling reasons. Firstly, Shanxi is a major coal-producing region, with numerous industries that significantly contribute to pollutant emissions such as sulfur dioxide, carbon monoxide, particulate matter, and PM_2.5_. Secondly, the province's distinctive topography, characterized by a series of discontinuous basins stretching from north to south, hinders the dispersion of pollutants and facilitates the formation of atmospheric secondary circulation, thereby exacerbating air pollution (Feng et al., 2021). Additionally, during the winter months, the widespread use of coal for centralized heating further intensifies air pollution in the area (Feng et al., 2021). Taking Taiyuan City, Shanxi Province as an illustrative case, the GBQ data reveals a rather concerning air quality situation. During the half-month research window from January 8th to January 20th, 2024, it is staggering to note that the number of days marred by severe air pollution exceeded half of the total days in that period. Expanding the scope to the entire month of January 2024, a mere 4 days witnessed the air quality attaining the excellent grade. The primary pollutant causing the pollution is PM_2.5_, followed by NO_2_ and PM_10_. Lastly, established relationships with several high schools in Shanxi Province facilitate the implementation of our survey-based research methodology.

### 3.2 Sample and procedure

This study adopts the daily diary research approach for two primary reasons. Theoretically, extensive research demonstrates that individuals' perceptions of air pollution and their emotional states fluctuate daily (e.g., Fehr et al., 2017; Koenen et al., 2019). The daily diary method is particularly adept at capturing these fluctuations in individuals' assessments of air pollution and their emotional responses. From a methodological standpoint, prior studies have established that the daily diary method not only accurately reflects the intrinsic changes within individuals but also illuminates the influence of time-invariant variables, such as personal traits, on these dynamic changes (Fehr et al., 2017). This makes it highly suitable for testing the proposed moderated mediation model in our study.

The statistical data provided by the Shanxi Provincial Department of Education indicates that, in 2023, a total of 65,100 teachers were employed in ordinary high schools throughout the entire province of Shanxi. By virtue of the personal connections we had meticulously cultivated, we ultimately succeeded in establishing contact with 213 EFL teachers hailing from 23 high schools within Shanxi Province. However, a portion of them exhibited reluctance to engage in the research. Eventually, the actual number of participants stabilized at 187. Subsequent to the removal of five questionnaires containing blatantly incorrect responses, the definitive sample size was ascertained to be 182, yielding a response rate of 85.4%. These high schools and their affiliated EFL teachers are distributed across diverse regions of Shanxi Province. To be more precise, Taiyuan City encompasses eight high schools, with a cumulative tally of 84 teachers; Changzhi City is home to five high schools, amounting to a total of 39 teachers; Jincheng City features four high schools, aggregating 33 teachers; Datong City comprises two high schools, with a combined count of 10 teachers; Xinzhou City contains two high schools, involving a total of eight teachers; and Yuncheng City incorporates two high schools, totaling eight teachers. The average age of participants is 36.2 years (*SD* = 4.53). Females make up ~69.2% of the sample. Notably, a significant majority (93.4%) of the participants hold postgraduate degrees or higher. The average professional experience among these English teachers is 6.2 years (*SD* = 2.17).

One week before initiating the main study, we sent a preliminary questionnaire and an automated system for testing WMC via email to each participant. The questionnaire included several key components: (1) A description of the study's objectives, along with ethical guidelines and a confidentiality statement; (2) Demographic questions to gather participant information; (3) A question designed to obtain participants' WMC: “Please fill in the blank with the numerical score you achieved after completing the working memory capacity test. My working memory capacity test result is ______.”; (4) A random code that participants were asked to remember for subsequent survey data matching. In line with recommendations from prior research (Khan et al., 2024), participants were required to complete a daily diary survey over 10 consecutive workdays. They need to rate their daily air pollution appraisals, mental efforts, and negative emotions. Participants were also instructed to record the random number assigned during the initial survey to ensure accurate data matching. We instructed participants to complete their diary entries each evening, ideally after their workday had concluded and just before retiring for the night. This timing was chosen to ensure that the entries captured the essence of their daily experiences and reflections on air pollution and their emotions. Our research team's consistent daily communication with the participants, coupled with the provision of a small token of appreciation as an incentive, significantly contributed to the success of our data collection efforts. As a result, we were able to gather 1,820 valid diary entries. The participants were diligent in their submissions, with the majority completing their entries by 7 pm daily, showcasing a high level of engagement and commitment to the study.

### 3.3 Measures

#### 3.3.1 Daily air pollution appraisals

EFL teacher daily air pollution appraisal was measured using Fehr et al. (2017)'s four items scale. Participants were required to rate the agreement level (*from 1*= *totally disagree* to *5*= *totally agree*) of the question “Today, the air pollution was (a) severe, (b) bad, (c) extreme, and (d) unbearable.”

#### 3.3.2 Daily mental efforts

We utilized a six-item self-reported scale originally developed by Krell (2017) to evaluate the daily mental efforts of EFL teachers. To ensure consistency with our research objectives, we modified original item “answer the task” to “finish the work.” Participants were required to express their level of agreement with each statement on a scale from *1* = *not at all* to *5* = *totally*.

#### 3.3.3 Daily negative emotions

The study employed a refined scale (Koenen et al., 2019) derived from Watson et al.'s (1988) Positive and Negative Affect Schedule to quantify the daily negative emotions of EFL teachers.

#### 3.3.4 Working memory capacity

We utilized the automated OSPAN task systems (Unsworth et al., 2005) to measure the WMC of EFL teachers. Our research team purchased one such system and distributed it to the participants. We asked them to perform the test independently and record their results in the preliminary questionnaire. The system involves participants initially verifying the correctness of a series of mathematical problems, followed by reading and memorizing a target word. This sequence is repeated across five sets, totaling 25 equation/word pairings. The evaluation of WMC is based on the number of words correctly recalled.

The questionnaire items of all the scales are listed in Appendix A.

#### 3.3.5 Control variables

According to previous research (e.g., Fehr et al., 2017; Galanakis et al., 2021), our study primarily controlled for variables that could potentially influence the main effect, including participants' demographic variables (age, gender, and tenure) and objective air pollution conditions. Age and tenure were measured by years, gender was measured using a binary variable (1 = female, 2 = male). The daily objective air pollution was obtained from the data recorded at the air quality monitoring station closest to the participants.

## 4 Results

### 4.1 Analytic approach

Given that our research data features a two-level nested structure (i.e., days nested within individuals), we have applied Multilevel Confirmatory Factor Analysis (MCFA) to assess the model's reliability and validity, and utilized two-level Hierarchical Linear Modeling (HLM2) to validate our hypotheses. The cross-level data analysis methods accurately partition the variance of daily observations into two components: between-individual variance and within-individual variance. This ensures that the estimation of within-individual effects and between-individual effects does not interfere with each other (Wang et al., 2013a). For the first level, we input daily data along with objective air pollution measurements, and for the second level, we input demographic information and WMC of teachers.

To investigate within-individual mediation effects and cross-level moderation effects, we employed the Monte Carlo Analysis available in the open-source R software. Monte Carlo analysis can generate asymmetric confidence intervals (CIs) around the observed indirect effect by generating random samples from the parameter distributions (Fehr et al., 2017). The computed asymmetric CIs are applicable to the skewed sampling distributions of indirect effects. This means that Monte Carlo analysis can more accurately handle the special distribution of indirect effects in the multilevel model of our study, thus providing more reliable statistical inferences for our research results. Moreover, to enhance the clarity of the moderation effects, we performed slope analysis and crafted visual plots.

### 4.2 CMB, reliability, validity, and descriptive statistics

In our cross-sectional study, considering that the data were gathered from a single origin and with an identical method, there was a possibility of common method bias (CMB) emerging as a potential concern, as suggested by Podsakoff et al. (2003). To address this, Harman's (1967) single-factor test was implemented. When all the items were compelled into a solitary factor, it was found that this factor could account for a maximum of 39.2% of the variance, which is less than the 50% threshold. Consequently, it was determined that CMB did not pose a significant problem in our study.

The hypothetical model we proposed underwent MCFA. The findings revealed that the model with four variables—air pollution appraisal, mental effort, negative emotion, and WMC—exhibited optimal fit indices (χ^2^ = 296.93, df = 138, CFI = 0.96, NFI = 0.91, RMSEA = 0.04). This model's fit is markedly superior to those of the models with three, two, and one factor variables, suggesting a good discriminant validity of our data.

Table 1 displays the means, variances, Cronbach's α values, and correlations among the main variables. The results indicate that all variables exhibit Cronbach's α values above 0.85, demonstrating high scale reliability. Significant correlations are observed between air pollution appraisal and mental effort, air pollution appraisal and negative emotion, mental effort and negative emotion, as well as WMC and negative emotion, which preliminarily validate the hypotheses we proposed.

**Table 1:** Descriptive statistics and correlations between the main variables.

	**Mean**	***SD***	**1**	**2**	**3**	**4**
**Daily variables**						
1. Air pollution appraisal	3.75	1.22	(0.88)	0.27^**^	0.11^**^	
2. Mental effort	3.64	1.71	0.33^**^	(0.91)	0.40^**^	
3. Negative emotion	4.12	0.89	0.15^**^	0.45^**^	(0.95)	
**Individual variables**						
4. Working memory capacity	11.28	5.47	**−0.02**	**0.04**	−0.37^**^	(0.86)

Within-individual correlations are shown above the diagonal (*N* = 1,820). Between-individual correlations are shown in bold (*N* = 182). Cronbach's α of each variable is displayed on the diagonal in parentheses.

** *p* < 0.01.

### 4.3 Hypotheses testing

We initiated the examination of mental effort's mediating role by adhering to the three-step mediation analysis (Baron and Kenny, 1986). As detailed in Table 2, Model 2 and Model 3 confirm that air pollution appraisal has a significant positive influence on mental effort (γ = 0.22, *p* < 0.01) and negative emotion (γ = 0.19, *p* < 0.01), respectively. When air pollution appraisal and mental effort are included in the model with negative emotion as the dependent variable, mental effort is found to have a positive effect on negative emotion (γ = 0.31, *p* < 0.01), while the effect of air pollution appraisal on negative emotion is notably reduced from 0.19 to 0.08. This reduction in effect size upon the inclusion of mental effort in the model supports the notion of partial mediation by mental effort.

**Table 2:** HLM results on variables.

	**Mental effort**		**Negative emotion**		**Negative emotion**		
	**Model 1**	**Model 2**	**Model 3**	**Model 4**	**Model 5**	**Model 6**	**Model 7**
Intercept	3.61^**^	3.61^**^	4.07^**^	4.07^**^	4.09^**^	4.08^**^	4.08^**^
**Level-1 variables**							
Objective air pollution	0.12^*^	0.11^*^	0.09^*^	0.09^*^	0.09^*^	0.08^*^	0.08^*^
Air pollution appraisal		0.22^**^	0.19^**^	0.08^*^			
Mental effort				0.31^**^		0.19^**^	0.14^**^
**Level-2 variables**							
Age	−0.08	−0.07	−0.06	−0.04	−0.06	−0.05	−0.05
Gender	0.02	0.01	0.02	0.01	0.02	0.02	0.01
Tenure	−0.05	−0.03	−0.01	−0.01	−0.01	−0.00	−0.00
Working memory capacity						−0.30^**^	−0.28^**^
**Cross-level interaction**							
Mental effort × working memory capacity							−0.12^**^
*e^2^*	0.33	0.31	0.22	0.21	0.22	0.20	0.20
*r^2^*	0.65^**^	0.63^**^	0.71^**^	0.70^**^	0.82^**^	0.78^**^	0.77^**^

e^2^and r^2^represent the within-individual and the between-individual variance in the variable.

* *p* < 0.05,

** *p* < 0.01.

To further delineate the indirect effect of air pollution appraisal on negative emotion through mental effort with greater precision, this study employed a Monte Carlo Analysis. We conducted 20,000 replications, consistent with previous literature (Barnes et al., 2015), to determine the confidence interval for the indirect effect. The results indicate a significant indirect effect of 0.08 [CI (0.005, 0.031)], which does not include zero. In conclusion, Hypothesis 1 was supported.

To investigate the cross-level moderating effect of WMC, we incorporated mental effort, WMC, and their interaction term into a regression model with negative emotion as the dependent variable. According to the results from Model 7 in Table 2, the interaction term is statistically significant (γ = −0.12, *p* < 0.01), confirming that WMC serves as a moderator in the relationship between mental effort and negative emotion.

**Figure 2:**
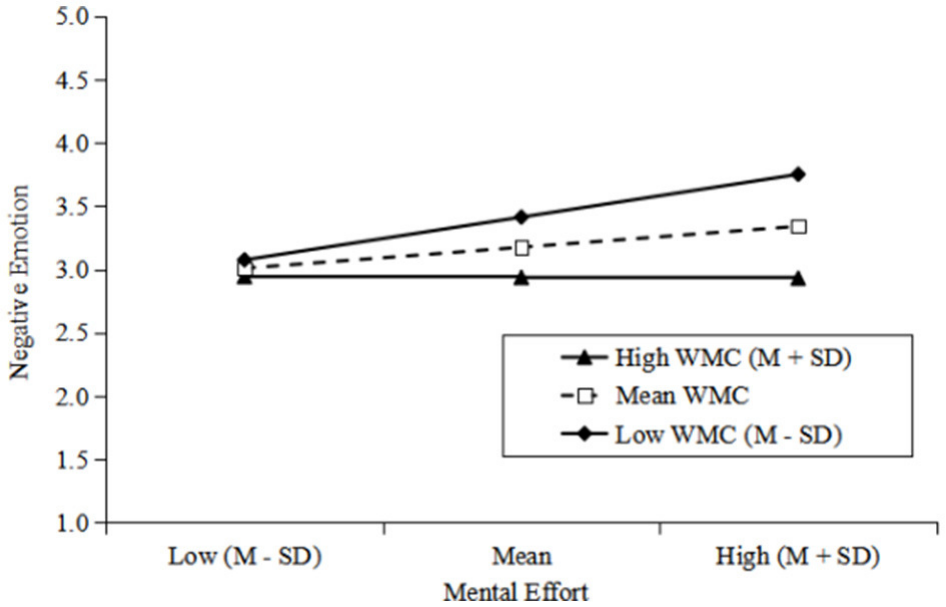
The moderating effect of WMC. Low refers to 1 *SD* below the mean. High refers to 1 *SD* above the mean.

For a more precise depiction of this moderating effect, we conducted a slope analysis and illustrated the moderation effect in Figure 2. The findings indicate that when WMC was high or medium (1 *SD* above the mean or equal the mean), the relationship between mental effort and negative emotion was nonsignificant (*B* = 0.04, *ns*; *B* = 0.07, *ns*). Conversely, at low levels of WMC (1 *SD* below the mean), the relationship is significant (*B* = 0.36, *p* < 0.01). Therefore, Hypothesis 2 was validated.

To test the moderating mediation hypothesis, we conducted Monte Carlo Analysis following the procedures as explained above. The results showed in Table 3 suggest that when WMC was high (1 *SD* above the mean), the conditional indirect effect was nonsignificant [*B* = 0.01, CI (−0.01, 0.02)]. However, when WMC was low (1 *SD* below the mean), the conditional indirect effect was significant [*B* = 0.11, CI (0.03, 0.19)]. In addition, the difference between these two effects was significant [Δ*B* = 0.10, CI (0.01, 0.16)], thereby supporting H3.

**Table 3:** Moderated mediation results.

**Moderator levels**	**Conditional indirect effect**	**95% CI**	
		**Lower**	**Upper**
High working memory capacity	0.01	−0.01	0.02
Low working memory capacity	0.11	0.03	0.19
Difference	0.10	0.01	0.16

Low refers to 1 SD below the mean. High refers to 1 SD above the mean.

## 5 Discussion

This study explores how EFL teachers' appraisal of air pollution impacts their emotions, utilizing a moderated mediation model that incorporates mental effort as the mediator and WMC as the moderator. Conducted through a daily diary survey of high school English teachers in Shanxi Province, China, our research confirmed all proposed hypotheses. Results indicate that ELF teachers' air pollution appraisal significantly increase their negative emotion which is mediated by mental effort. Furthermore, WMC serves as a negative moderator in the relationship between mental effort and negative emotions, as well as in the “air pollution appraisal–mental effort–negative emotion” pathway. This suggests that these relationships are less pronounced for teachers with higher WMC compared to those with lower WMC. This study not only contributes significant theoretical insights to the related literature but also provides practical guidance on how to cope with emotional challenges posed by environmental stressors, particularly air pollution.

### 5.1 Theoretical implication

This study contributes theoretically in several ways. Firstly, it identifies natural environmental factors, such as air pollution, as significant antecedents that influence ELF teacher emotions. This insight effectively broadens the existing body of literature on EFL teacher emotions and air pollution appraisal. While the interplay between natural environments and individual psychology has been noted in fields like business administration and psychology (e.g., Fehr et al., 2017; Khan et al., 2024), the educational and linguistic communities have been relatively slower to recognize the significance of these factors. Existing research on the determinants of EFL teacher emotions has been primarily centered on social factors such as individual, interpersonal, and organizational influences (Han et al., 2023). For example, some studies focus on the impact of various factors on EFL teachers' emotions from their own perspectives, including teaching abilities (Song, 2021), educational backgrounds (Kocabas-Gedik and Hart, 2021), and teaching objectives (Kang, 2022). Other research examines how teacher-student interactions and relationships with colleagues (Alvandi et al., 2015) influence their emotions. Additionally, some studies explore these impacts from a broader perspective, such as the social environment and cultural background (Hayik and Weiner-Levy, 2019). However, there is currently a lack of research on how the physical and natural environment affects teachers' emotions. As highlighted in prior research, the existing studies examining the factors influencing teacher emotions are relatively rudimentary, “future research should comprehensively investigate this area to attain a more nuanced understanding of the complexity inherent in teacher emotions” (Chen, 2021, p. 349).

In response to this call for research, we introduce air pollution appraisal as an independent variable, proposing that EFL teachers facing air pollution might experience severe negative emotions due to physical discomfort, decreased cognitive abilities, and increased cognitive load. This aligns with findings from other disciplines (e.g., Jones and Whitehouse, 2021; Khan et al., 2024). Furthermore, to our knowledge, while some studies in education and linguistics have explored the effects of air pollution on teachers' physical health and performance (Balakrishnan and Tsaneva, 2021), our research is pioneering in examining how natural environments like air pollution influence EFL teacher emotions. Thus, our study not only responds to scholarly calls for deeper investigations into the determinants of teacher emotions but also breaks new ground in studying the psychological and emotional impacts of environmental factors on EFL educators.

Secondly, leveraging Cognitive Load Theory, our research introduces mental effort acts as a mediator between air pollution appraisal and EFL teachers' negative emotions. Prior studies in education and linguistics have predominantly examined straightforward relationships between variables, often overlooking the mediating mechanisms underlying these associations (Han et al., 2023). Meanwhile, although a link between teachers' cognitive processes and emotional states has been acknowledged, empirical studies remain scarce (Xu, 2018). Additionally, while cognitive load is a crucial concept within educational field, its application in examining the emotional impacts on teachers has been limited, with prior research focusing predominantly on how emotions influence cognitive load (Chang and Chen, 2024). Our research departs from earlier studies by adopting a cognitive load perspective and proposing a mediation model where air pollution not only diminishes EFL teachers' cognitive and respiratory capacities, compelling them to exert more mental effort in teaching tasks but also depletes their cognitive resources, thus exacerbating their mental effort. This increased mental exertion can lead to cognitive dissonance and diminished emotional regulation capabilities, resulting in heightened anxiety, unrest, annoyance, and fear among teachers.

Theoretically, our study significantly contributes by mapping out the full causal pathway from air pollution assessment to the elicitation of negative emotions in EFL teachers, clearly showing how external environmental factors can escalate cognitive burdens and deplete cognitive resources, resulting in negative emotional states. This echoes the findings of previous studies that air pollution impacts individual cognition functions and performance (Clifford et al., 2016; Zhang et al., 2018). It also clarifies that an increase in cognitive load can precipitate emotional changes in EFL teachers, thus confirming that cognitive load and teacher emotions interact dynamically. In essence, by providing empirical evidence that cognitive load can precipitate negative emotions, our study not only extends the application of cognitive load theory in EFL teaching but also enhances understanding of how air pollution assessment leads to negative teacher emotions, deepening insights into the mechanisms by which air pollution exerts its effects.

Thirdly, our study introduces a boundary condition within the aforementioned mediation relationship, specifically that the dynamic process by which air pollution appraisal increases EFL mental effort, leading to greater negative emotions, varies according to the individual capabilities (i.e., WMC) of EFL teachers. This discovery is vital, as previous research primarily focused on identifying moderators to reduce teachers' negative emotions through external sources like social support and interpersonal relationships (e.g., Ghasemi, 2022; Wang et al., 2013b), or through internal emotional regulation factors, such as emotional intelligence (e.g., Yin, 2015). However, few studies have explored how variations in cognitive abilities might influence emotional responses. Some scholars have indicated that individuals' WMC can enhance their ability to suppress negative emotional expressions (Schmeichel and Demaree, 2010). Leveraging Cognitive Load Theory, our research expands on these insights by suggesting that EFL teachers with higher WMC can effectively use their enhanced cognitive resources to counteract the cognitive depletion caused by air pollution appraisal and the adverse effects of increasing mental effort, thereby reducing the incidence of negative emotions. Consequently, this study integrates cognitive load, cognitive abilities, and EFL teachers' emotional changes into a cohesive theoretical framework, empirically confirms the proposed moderating effect and significantly advancing our understanding of the interplay between EFL teachers' cognition and emotion.

### 5.2 Practical implication

This study presents important practical implications. Firstly, to mitigate the impact of air pollution on EFL teachers' emotions, schools should develop new policies or human resource management strategies that enable teachers to effectively counteract the adverse effects of air pollution. Essential measures include providing comprehensive hardware and software support to both teachers and students, ensuring that English teaching can be conducted remotely during severe pollution days. Additionally, schools could implement flexible scheduling based on air quality, allowing for adjustments to class times during extreme pollution events. Moreover, schools should supply N95 masks and air purifiers to teachers and students to minimize the educational disruptions caused by poor air quality.

Secondly, our research underscores the importance of an EFL teacher's WMC in managing the negative emotions triggered by air pollution. Therefore, schools might consider incorporating WMC assessments into their hiring processes, giving preference to candidates with higher WMC when qualifications are similar. Additionally, since research suggests that WMC can be developed through training, schools should also evaluate the WMC of existing teachers and provide targeted cognitive resilience training. Techniques such as complex-span training and music training have been shown to enhance WMC (Harrison et al., 2013; Lee et al., 2007). By improving teachers' cognitive resilience, schools can better equip their staff to handle the challenges posed by air pollution. It should be emphasized that this does not mean that simply relying on the assessment of working memory ability can completely resolve the issue of teachers' negative emotions. In fact, including it as one of the recruitment considerations aims to enhance the overall psychological resilience of the teaching staff in complex environments.

### 5.3 Limitation and future directions

Although our study offers significant theoretical and practical implications, it is not without limitations. Firstly, the sample size of our research is relatively limited and primarily comprises high school English teachers from Shanxi Province, China. In light of prior research indicating that people's awareness and perceptions of environmental pollution can markedly differ across various regions and pollution intensities, we must exercise caution when extrapolating our findings. Notably, as demonstrated by Wei et al. (2016), residents in cities with relatively lower pollution levels tend to be more sensitive to air quality concerns. Consequently, we propose to replicate this study in diverse geographical locations and under varying pollution scenarios, while also augmenting the sample size of the investigation. By doing so, we aim to widen the applicability and relevance of our research conclusions. Secondly, our data collection method involved having participants record daily diaries every evening. This cross-sectional approach may introduce recall bias or skew the data toward end-of-day reflections. In future research, more frequent data collection throughout the day should be considered to present a more accurate and dynamic picture of participants' experiences and emotional states. In addition, to more clearly describe the causal relationship between air pollution assessment and teachers' negative emotions, future research could consider tracking the impact of macro-panel air pollution data on emotions over a period of several years. Alternatively, experimental research could also be carried out. At the same time, since there have been some relevant empirical studies in this field, the use of meta-analysis methods to investigate the causal relationship between the two could also be considered.

Thirdly, our findings indicated that mental effort served as a partial mediator in the “air pollution appraisal–negative emotion” relationship, suggesting the potential existence of other mediating mechanisms that could further elucidate the relationship. Future research could investigate additional mediators, such as those derived from self-control theory (Fehr et al., 2017). Finally, while our study focused on individual-level moderating variables, previous research has highlighted the potential moderating effects of environmental and interpersonal variables (Han et al., 2023). Investigating these additional moderating variables in future studies could help elucidate the full complexity of the “air pollution appraisal–mental effort–negative emotion” relationship, providing a more holistic view of the factors at play. Fourthly, another limitation of this study is the lack of a falsification test to assess the robustness of the findings. Future research could incorporate data on air pollution from more distant cities to evaluate whether the observed negative effects persist in cases where direct impacts might be less plausible. This approach would help strengthen the validity of the conclusions and provide a more comprehensive understanding of the relationship.

Finally, this study has not sufficiently addressed the lagged effects of air pollution. Lagged effects are prevalent in phenomena related to air pollution, as evidenced by delayed impacts on outcomes such as suicide tendencies, spontaneous abortions, and other health-related issues (Davoudi et al., 2021; Zhou et al., 2022). By focusing primarily on the immediate relationship between air pollution and teachers' emotions, this research may have overlooked significant long-term and cumulative effects. To address this limitation, future studies could adopt a longitudinal research design to monitor air pollution levels and relevant outcomes over an extended period. Such an approach would allow for the identification and quantification of lagged effects, providing a more comprehensive understanding of the complex relationship between air pollution and English teachers' emotions.

## 6 Conclusion

In the present study, we delved into the underlying mechanism through which EFL teachers' air pollution appraisal impact their negative emotions. Grounded in the cognitive load theory, our findings revealed that air pollution appraisal has the potential to augment the mental effort of EFL teachers, subsequently triggering a greater prevalence of negative emotions. Notably, this effect is modulated by individual characteristics, i.e., working memory capacity. When teachers possess a relatively high working memory capacity, the adverse influence tends to be mitigated; conversely, a lower working memory capacity exacerbates the negative impact. This research not only significantly broadens the scope of existing studies concerning the nexus between air pollution and teachers' emotions but also offers practical guidelines for educational institutions in their efforts to address environmental pollution challenges.

## Data Availability

The original contributions presented in the study are included in the article/supplementary material, further inquiries can be directed to the corresponding author.

## Appendix

**Table A1:** Measures of constructs.

**Construct**	**Source**
*Daily air pollution Appraisal*	Fehr et al. (2017)
Today, the air pollution was (a) severe, (b) bad, (c) extreme, and (d) unbearable	
*Daily mental efforts*	Krell, 2017^*^represent reversed items
1. The work was difficult to finish	
2. The contents of the work were complicated	
3. I haven't taken particular trouble with the reply to the work^*^	
4. The work was easy to work on^*^	
5. I have made an intellectual effort when finishing the work	
6. I haven't tried hard to finish the work^*^	
*Daily negative emotions*	Koenen et al. (2019) and Watson et al. (1988)
In the list below, you see several emotions which you may have experienced during the day. Please mark for each emotion to what extent you have felt that emotion from *1 = almost not* to *5* = *quite strong:* anger, guilt, helplessness, irritation, sadness, and tension.	
*Working memory capacity*	Unsworth et al. (2005)
OSPAN task systems from the below link: https://www.millisecond.com/download/library/v7/ospan/automatedospan/automatedospan.web	
